# Role of Cyclodextrins and Drug Solid State Properties on Flufenamic Acid Dissolution Performance from Tablets

**DOI:** 10.3390/pharmaceutics14020284

**Published:** 2022-01-26

**Authors:** Francesca Maestrelli, Marzia Cirri, Enrico De Luca, Diletta Biagi, Paola Mura

**Affiliations:** 1 Department of Chemistry “U. Schiff”, University of Florence, Via Schiff 6, Sesto Fiorentino, 50019 Florence, Italy; francesca.maestrelli@unifi.it (F.M.); enrico.deluca1187@gmail.com (E.D.L.); paola.mura@unifi.it (P.M.); 2 Menarini Manufacturing Logistic and Services s.r.l. (AMMLS), Via dei Sette Santi 1/3, 50131 Florence, Italy; dbiagi@menarini.it

**Keywords:** flufenamic acid, cyclodextrins, tablet, solid state, dissolution

## Abstract

Flufenamic acid (FFA) is a non-steroidal anti-inflammatory drug characterised by a low solubility and problems of variable dissolution rate and bio-inequivalence. Different FFA batches, obtained by different suppliers, showed different powder characteristics (particle size, shape and surface properties) that may affect its dissolution behaviour from solid dosage forms. Aim of this work was the improvement of FFA solubility and dissolution rate by the use of cyclodextrins (CDs) and the obtainment of an effective tablet formulation by direct compression. Several CDs have been tested, both in solution and in solid state and several binary systems drug-CDs have been obtained with different techniques, with the scope to select the most effective system. Grinding technique with randomly methylated-β-cyclodextrin (RAMEB) was the only one that allowed the complete drug amorphization, together with the highest improvement in drug dissolution rate, and was then selected for tablets formulation. Conventional and immediate release tablets were obtained and fully characterised for technological properties. In both cases an improved and well reproducible drug dissolution performance was obtained, independently from the FFA supplier and thus no more affected by the differences observed between the original FFA crystalline samples.

## 1. Introduction

Oral route is the main way of administration of drugs, especially by the use of tablets. The bioavailability of an orally administered drug mainly depends on its solubility in the gastrointestinal tract fluids and its permeability through the biological membranes [1]: its dissolution in gastrointestinal fluids is a prerequisite for enabling a drug to permeate through the biological membranes and thus reach the systemic circulation and exploit its therapeutic action. However, unfortunately, a very large number of drugs are sparingly soluble or practically insoluble in water, and the number of poorly water soluble drug candidates has increased in the last years, achieving about 80% [2]. Therefore, poor solubility continues to represent a major obstacle in the successful development of new medicines. For this reason, the development of new drug-delivery strategies enabling effective formulations of such drugs, able to improve and make more reproducible their bioavailability and overcome problems of bio-inequivalence among pharmaceutically equivalent dosage forms, or even among different production batches, is of great interest [3]. Solubility and dissolution rate of a drug not only depend upon its particle size, solid-state (crystalline or amorphous, crystal habitus, anhydrous or solvated form, polymorphic form), and physicochemical properties (partition coefficient, lipophilicity, wettability, etc.), but also upon the properties of the dosage form used to administer the drug. For example, in the case of tablets, it is important to carefully evaluate the properties of the excipients used (wettability, particle size, solid state…) and the conditions and technological processes used for their production, and the choice of the best formulation strategy is far from a straightforward process [4]. Flufenamic acid (FFA) is a non-steroidal anti-inflammatory drug derivative of N-phenylanthranilic acid, well-known for its analgesic, anti-inflammatory, antipyretic and analgesic action. Furthermore, it has recently been reported that FFA also possesses other interesting activities such as ionic channels modulation of cardiomyocytes, neurons and smooth muscle cells [5,6]. FFA is categorised according to the Biopharmaceutical Classification System as a class II drug (low solubility, high permeability). Issues regarding high bioavailability variability after oral administration of FFA commercial capsules or tablets have been reported [7,8,9]. Several technological processes used during manufacturing of solid dosage forms (e.g., grinding, use of solvents during granulation, compression) can modify drug solid state properties and then affect its dissolution rate, rendering the production process not well reproducible and giving rise to problems of non-bioequivalence between different batches [10]. In addition, FFA has nine proposed polymorphs, even though Forms I and III, enantiotropically related, are the most stable and the only ones existing at ambient temperature [11]. The influence of polymorphism on bioavailability of poorly hydro-soluble drugs has been amply demonstrated [12]. A different intrinsic dissolution rate was observed between two FFA samples from two different firms, even using the same powder granulometric fraction [13]. An enhancement of FFA solubility and dissolution rate could be particularly useful to overcome all the issues associated with its poor-solubility, leading to an increased and more reproducible bioavailability and enabling the development of more effective and robust solid dosage forms of the drug, strongly reducing risks of bio-inequivalence issues. Cyclodextrins (CDs) are cyclic oligosaccharides able to form inclusion complexes with a variety of lipophilic drugs, leading to an improvement in their solubility and stability [14,15]. Previous studies showed the solubilising ability toward FFA of β-CD [16,17] and hydroxypropyl-β-CD [18,19]. Moreover, the drug-cyclodextrin binary systems preparation technique can strongly affect drug dissolution properties, since some methods can be more effective than others in improving the interactions with the cyclodextrins and/or reducing drug particle size (i.e., supercritical antisolvent micronisation, grinding, freeze drying and spray-drying) [20,21,22]. Based on all these premises, in the present work it was considered of interest to test and compare the complexing and solubilising properties towards FFA of the three natural CDs (α, β and γCD) and of some β-CD derivatives (hydroxypropyl-, sulfobutylether- and randomly methylated-β-CD), and to investigate the effect of the preparation method of the corresponding drug-CD solid systems (physical mixing; kneading, grinding, co-evaporation, co-lyophilisation) on FFA dissolution rate. Interactions between FFA and the different CDs have been in depth investigated, both in solution and in solid state, in order to properly select the most effective CD and the best binary system preparation method to maximise and make more reproducible the drug dissolution behaviour, so as to overcome the above described issues. Once selected the most effective CD and the best preparation technique of FFA-CD binary system, two types of tablets have been developed: immediate release tablets (IRTs) rapidly releasing the drug, suitable for the treatment of acute diseases; and conventional tablets (CTs) with enough hardness suitable for enteric coating and to be used for the treatment of chronic diseases, requiring a reduction of side effects (e.g., gastrointestinal aggressiveness) and a slow gradual release. All tablets were fully characterised by classic tests (drug content, mean weight, diameter, thickness, friability, hardness, disaggregation time and dissolution rate). Moreover, the effect of using FFA powder samples obtained from two different suppliers was also evaluated, considering the unexpected differences in their intrinsic dissolution rate previously detected as well as in the different wettability of the surface of their compacts [13]. For this reason, possible differences in wettability of the obtained tablets were also investigated, since they could be an additional factor responsible for differences in drug dissolution rate. This study should allow us to get more insight about the previous unexpected results and, above all, to provide a suitable tool for obtaining a better and more reproducible dissolution behaviour of this drug, thus overcoming its frequent bioinequivalence issues.

## 2. Materials and Methods

### 2.1. Materials

Two different batches of flufenamic acid (2-{[3-(trifluoromethyl) phenyl] amino} benzoic acid), FFA) were used: a batch donated by S.I.M.S. (FFA SIMS) (Florence, Italy) (purity 99.97% [13]) and another batch provided by TCI EUROPE N.V. (FFA TCI) (Zwijndrecht, Belgium) (purity 99.99% [13]). CDs were from Merck Life Science S.r.l. (Milan, Italy); βCD and HPβCD (MS 0.86) were a gift from Roquette (Lestrem, France); sulfobutylether-βCD (SBEβCD) was donated by Cyclolab (Budapest, Hungary) and randomly methylated-βCD (RAMEB) by Wacker-Chemie (Munich, Germany). Tablet excipients: partially pregelatinised starch was from Roquette (Lestrem, France); spray-dried lactose from Meggle Group (Wasserburg, Germany), magnesium stearate and polyvinylpyrrolidone (PVPK90) from Merck Life Science S.r.l. (Milan, Italy). All other chemicals were of analytical reagent grade.

### 2.2. Phase Solubility Studies

Phase solubility studies were carried out by adding an excess amount of FFA (100 mg) to 10 mL of phosphate buffer pH 6.8 containing increasing concentrations of CD (0–25 mM or 0–12.5 mM in case of βCD). The vials were sealed and electromagnetically stirred (750 rpm) at constant temperature (37 °C) until equilibrium (3 days). An aliquot of solution was then withdrawn with a filter syringe (pore size 0.45 μm) and spectrometrically assayed for drug concentration at 290.8 nm (UV/V is 1900 Shimadzu Spectrophotometer, Tokyo, Japan). The presence of CD did not interfere with the spectrophotometric assay of FFA. Each experiment was performed in triplicate (coefficient of variation (CV) <2.5%). The apparent 1:1 stability constant K_1:1_ and the complexation efficiency (CE) of the complexes were calculated as follows [23]:

K1:1 = m/So (1 − m)

CE = m/(1 − m)

where m is the slope of the straight lines of the phase solubility diagrams, and So the drug solubility in the medium.

### 2.3. Preparation Methods of Drug: CD Solid Binary Systems

Drug–CD solid systems were prepared in equimolar ratio, based on the results of phase-solubility studies, indicative of the formation of 1:1 (mol/mol) FFA–CD complexes. Five different methods were used for their preparation. Physical mixtures (PM) were obtained by tumble mixing for 15 min equimolar amounts of the respective components (75–150 µm sieve granulometric fraction); co-ground systems (GR) were prepared by ball-milling for 30 min at 24 Hz physical mixtures in a high-energy vibrational micro-mill (Mixer Mill MM 200 Retsch, GmbH, Dusseldorf, Germany); kneaded products (KN) were prepared by wetting the physical mixture with a small volume of an ethanol–water 1:1 (*v*/*v*) solution and kneading thoroughly with a pestle to obtain a homogeneous slurry and continuing until the solvent was completely removed, and then drying the products in a vacuum desiccator for 24 h to remove traces of solvent; co-evaporated products (COE) were obtained by co-evaporation of equimolar FFA/CD ethanol–water 1:1 (*v*/*v*) solutions in a rotary evaporator at 70 °C and drying the resulting products in a vacuum desiccator for 24 h to remove traces of solvents; co-lyophilised products (COL) were prepared by freeze-drying at 50 °C and 1.3 × 10^−2^ mmHg equimolar drug–CD aqueous solutions (Freeze Dryer Lyovac GT 2 Leybold Heraeus, Köln, Germany).

### 2.4. Characterisation of Drug: CD Binary Systems

Binary systems were characterised by differential scanning calorimetry (DSC) and X-ray powder diffractometry (XRPD). Thermal analysis was performed with a Mettler TA4000 Star^e^ Software calorimeter equipped with a DSC25 cell (Mettler Toledo, Switzerland). Briefly FFA, CDs and the obtained binary systems were weighed with a Mettler MX5 Microbalance (5–10 mg), and scanned in Al pans pierced with a perforated lid under static air with a heating rate of 10 °C/min, from 30 °C to 200 °C. The instrument was calibrated with standard Indium samples for temperature and heat flow. Drug melting enthalpy values were obtained by the area under the curve of the drug melting endotherm, calculated by the Software of the Mettler TA4000 calorimeter, and referred to the weight of FFA in each sample. Data are the mean of three determinations.

For each sample, the percentage of residual crystallinity (% Rc) of drug was calculated as follow:

% Rc = (ΔH sample/ΔH FFA) × 100

A Bruker D8-advance (Billerica, MA, USA) X-ray powder diffractometer was employed for recording the diffraction patterns of drug and drug–CD binary systems. The parameters were: Cu Kα radiation = 1.54056 Å, voltage 40 kV, current 40 mA and 2θ over a 5–30° range at a scan rate of 0.05°/s with 0.02° increments and a step time= 0.8 s/step. A Si (Li) solid-state (Sol-X^®^) was used as a detector, and C/Ni Goebel-Spiegel mirrors in the incident beam were used as monochromators. All samples were examined at room temperature.

In vitro dissolution studies of the prepared solid products were performed using a modified dispersed amount method [13]. Briefly, a sample amount corresponding to 200 mg of FFA was added to 75 mL of phosphate buffer (pH 6.8) at 37 °C and gently stirred (100 rpm) by a three-blade propeller. At predetermined time intervals, aliquots of 3 mL were withdrawn with a syringe-filter (0.45 µm Millipore membrane filter) and immediately replaced with the same volume of fresh dissolution medium, thermostated at the same temperature. The drug amount in the samples was spectrophotometrically assayed as described previously. A correction was applied for the cumulative dilution. All experiments were repeated three times (CV < 2.5%). All dissolution studies were performed at 37 °C and pH 6.8 in order to better compare the results.

### 2.5. Tablets Preparation

Tablets were prepared by direct compression (2.5 tons for 3 min) of the powders with a hydraulic press. The tablets were prepared using the selected drug-CD binary system FFA-RAMEB GR 1:1 mol/mol (50 mg FFA and 233 mg of RAMEB) and, as comparison, the simple PM and FFA alone, as references. For immediate release tablets (IRTs) the excipients used were spray-dried lactose (100 mg), starch (50 mg) and magnesium stearate (0.1%). For conventional tablets intended for enteric coating (CTs) a dry binder (PVPK90, 50 mg) was added to the previous formulation. Conventional tablets were prepared using both FFA SIMS and FFA TCI samples.

### 2.6. Characterisation of Tablets

All tablets were characterised for drug content, mean weight, diameter, thickness, friability, hardness, disaggregation time, according to official F.U. XII and Ph. Eur. X. Dissolution studies of FFA from tablets were performed as described above for FLU-CD binary systems; experiments were repeated three times (CV < 4.0%). Dissolution efficiency at 60 min (DE60) was calculated from the area under the dissolution curve at time t (measured using the trapezoidal rule) and expressed as a percentage of the area of the rectangle described by 100% dissolution in the same time [24]. Conventional tablets were also characterised by a contact angle study, to investigate the possible role of tablet surface wettability in drug dissolution rate. A Pocket Goniometer PGX (Exa-marketing s.r.l., Torino, Italy) was used and the angle between a water droplet and the horizontal surface of classic tablets was measured. A dynamic acquisition was made, collecting photos at equal time intervals (around 3 ms) during a total time of 30 s, starting from the moment the droplet fell on the tablet surface, determining the variation of the contact angle as a function of time. True density of the tablets was determined at 25 °C in Helium using a Gas Pycnometer Ultrapyc 5000 by Anton Paar Italia s.r.l. (Rivoli, Italy).

## 3. Results

### 3.1. Phase-Solubility Studies

**Figure 1:**
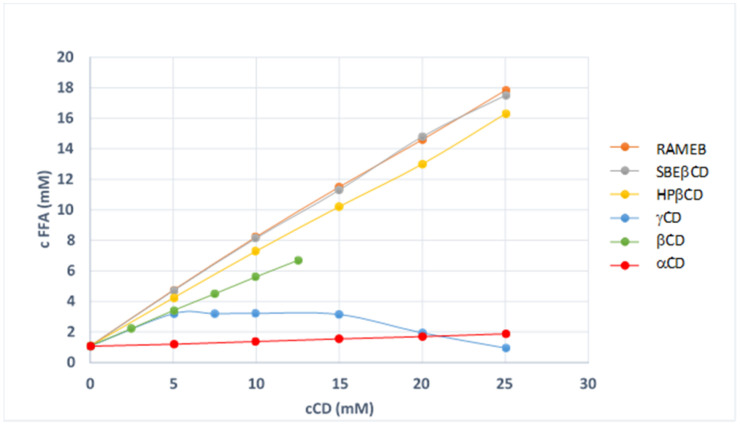
Phase solubility diagrams of FFA with different CDs at 37 °C in pH 6.8 phosphate buffer.

In order to evaluate and compare the complexing and solubilising abilities of the different CD towards FFA, phase solubility studies have been performed in phosphate buffer (pH 6.8) with the three natural Cds, α, β and γCD and three β-derivative CDs, RAMEB, HPβCD and SBEβCD. Since no differences were previously observed in the equilibrium solubility value of FFA obtained from SIMS or TCI suppliers [13], phase solubility studies were performed using only FFA from TCI. As shown in Figure 1, in case of all examined CDs (except for γCD) FFA solubility increased linearly with increasing CD concentrations, showing typical AL–type phase diagrams indicative of the formation of soluble complexes of probable 1:1 mol:mol stoichiometry [25]. On the other hand, in the case of γCD, a Bs–type diagram was found, indicating the formation of an insoluble inclusion complex.

As shown in Table 1, among natural CDs, αCD demonstrated a very low affinity for the drug, in fact a very low solubility enhancement was appreciable, probably due to the unsuitability of its cavity to accommodate the drug. On the other hand, βCD showed the highest complexing and solubilising ability towards FFA, suggesting that its cavity had the most suitable size to allow an effective drug accommodation inside. Moreover, the presence of substituents enabled to markedly increase the performance of βCD, which was in the order SBEβCD ≅ RAMEB > HPβCD >> βCD thus indicating a higher affinity of βCD derivatives toward FFA compared to the native one.

### 3.2. Preparation and Solid-State Characterisation of Drug-CD Binary Systems

Binary systems of FFA with the different examined CDs were then prepared with several methods (physical mixing, kneading, co-grinding, co-evaporation and co-lyophilisation), and carefully characterised, in order to evaluate the effect of the preparation technique on the properties and performance of the obtained products. This is particularly important in the case of FFA, since, as above discussed, it may exist in two main polymorphic Forms (I and III), enantiotropically related, being Form I the metastable one below the transition temperature (42 °C) [10], and polymorphic transition could happen during sample preparation by the different methods. On the other hand, since previous studies showed that FFA SIMS and FFA TCI powders were both in the Form I and superimposable results were observed in their dissolution rate by the dispersed amount method [13], the initial screening for the choice of the best CD and preparation method was performed using only FFA TCI.

Results of DSC studies are summarised in Table 2 in terms of FFA melting temperature, fusion enthalpy and % residual crystallinity. Some representative thermograms are shown in Figure S1 Supplementary.

The melting endotherm of pure FFA peaked at 135.6 °C (TCI) or at 135.5 °C (SIMS), confirming that it was provided by both suppliers in the polymorphic Form I.

The drug melting peak was clearly evident in all binary systems with native crystalline βCD, even though a progressive significant decrease of fusion enthalpy indicating loss of drug crystallinity was observed when passing from the PM to KN, COE, GR and COL products (from 92.5% Rc of PM up to 24.4 and 20.7 for GR and COL products, respectively).The same trend in enthalpy of fusion reduction, and then in decline of drug crystallinity was observed in the case of various FFA binary systems with the amorphous derivatives HPβCD and SBEβCD, but the effect was clearly more evident, achieving a % residual crystallinity in GR and COL products ranging from 14 to 10%. This result was considered indicative of establishing more effective drug-CD solid state interactions with such amorphous βCD-derivatives, and confirmed grinding and co-lyophilisation as the better preparation techniques to bring about their formation. On the other hand, this effect was even more marked for FFA products with RAMEB, which showed the highest complexing/amorphizing properties, giving rise to the complete disappearance of the drug melting peak in all binary systems except the simple PM.

It is also interesting to point out the shift of drug melting peak from 135 to about 125 °C observed in the case of COE products with βCD, HPβCD and SBEβCD, all obtained by evaporation of a hydroalcoholic solution. An analogous shift of drug melting peak from 135 to about 125 °C was previously observed also on pure drug samples obtained by recrystallisation from hydroalcoholic solution at 78 °C, and DSC and XRPD analysis proved that it was due to the formation of its polymorphic Form III [13]. Therefore, the formation of FFA form III in its COE products with the various CDs can be reasonably hypothesised. 

XRPD studies have been performed to confirm these results and exclude any possible influence of the DSC technique, since the thermal energy provided to the sample during the analysis could bring about the drug amorphization process in the presence of the amorphous carrier.

**Figure 2:**
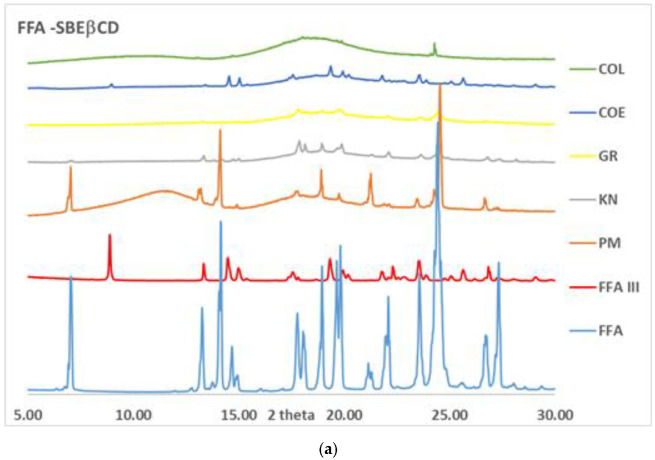

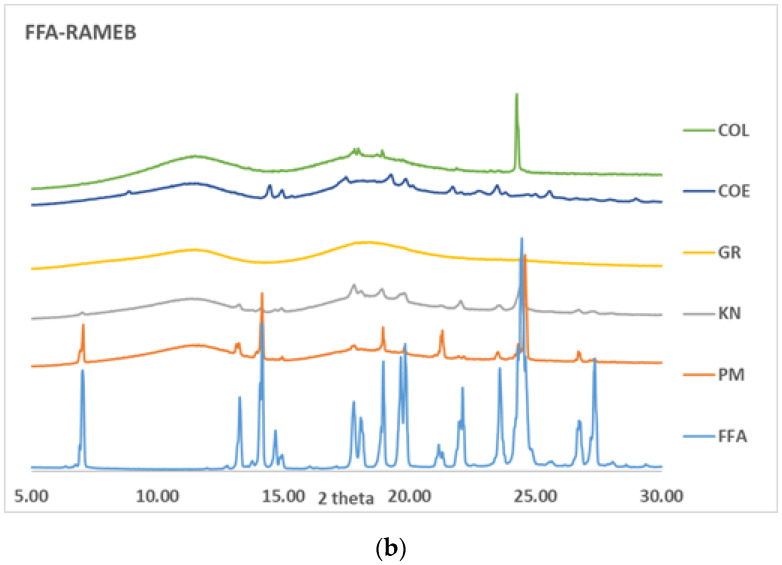
XRPD patterns of FFA binary systems with SBEβCD (**a**) and RAMEB (**b**) obtained by physical mixing (PM), kneading (KN), co-grinding (GR), co-evaporation (COE) and co-lyophilisation (COL).

XRPD diffractograms of the different binary systems of FFA with SBEβCD and RAMEB are reported in Figure 2 together with those of pure drug as Form I and Form III (obtained by recrystallisation from a hydro-alcoholic solution). As shown for the series of FFA systems with SBEβCD (Figure 2a), reported as an example since similar results were obtained for βCD and HPβCD series, the drug crystallinity peaks typical of Form I were well evident in the physical mixture, while in the other binary systems they were clearly reduced in intensity and number, but still detectable. Moreover, in the COE product the presence of the FFA Form III, above evidenced by DSC analysis, was confirmed by its typical peak at 9° 2 Θ, absent in Form I. In the Figure 2b is reported the series of FFA systems with RAMEB, where the complete drug amorphization observed by DSC analysis was confirmed only for the GR product, thus evidencing an artefact in the DSC results for KN, COE and COL products, due to the thermal technique. Moreover, in this case, the presence of FFA in the Form III was appreciable in the COE product.

The XRPD analysis of GR FFA-RAMEB repeated after 1 year from the sample preparation allowed to establish the maintenance over time of complete sample amorphization.

### 3.3. Dissolution Rate Studies of Binary Systems

**Figure 3:**
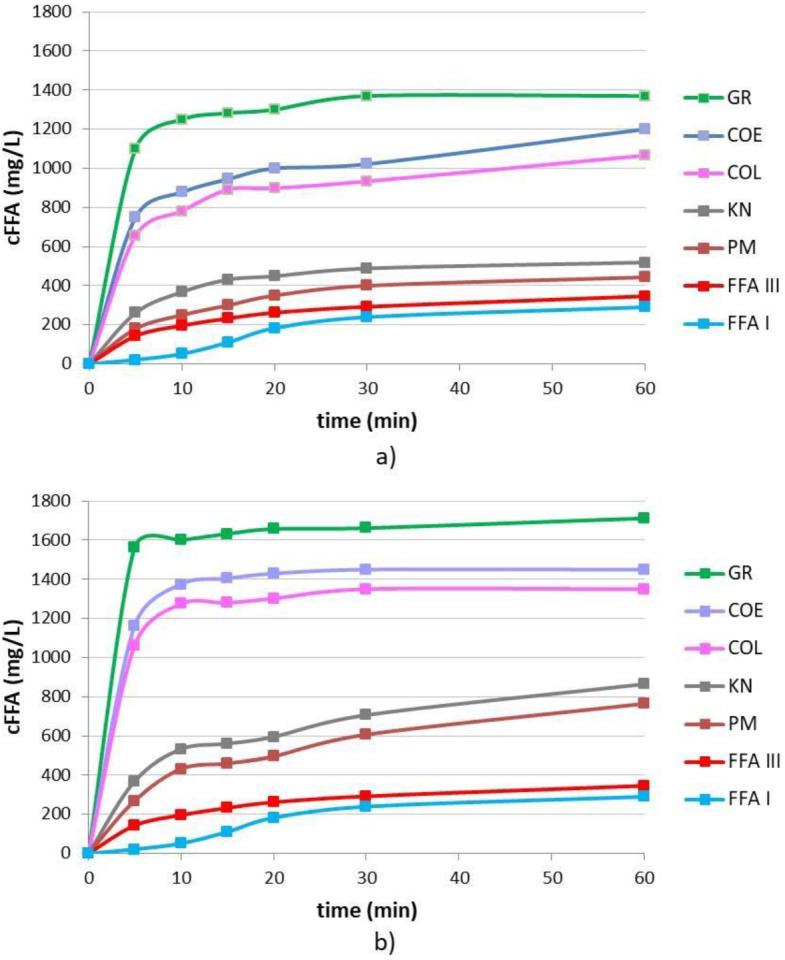
Dissolution curves of FFA alone (Form I) or from its binary systems prepared by physical mixing (PM), kneading (KN), co-lyophilisation (COL), co-evaporation (COE) or co-grinding (GR) with HPβCD (**a**) or RAMEB (**b**). The dissolution curve of FFA Form III is also shown.

The dissolution behaviour of all drug-CD binary samples has been evaluated and compared with that of drug alone as (Form I) (Figure 3). Almost superimposable dissolution profiles of sieved samples of FFA SIMS and FFA TCI were observed, confirming the result of the previous study [13]. The dissolution curve of FFA Form III (obtained by recrystallisation of Form I from a hydro-alcoholic solution) has been also reported for comparison purposes.

In Figure 3a the dissolution profiles obtained with the series of FFA-HPβCD binary systems were reported as an example, since similar results were obtained also with CD and SBEβCD series. All these CDs demonstrated a good ability to improve FFA dissolution properties and a good correlation between amorphization and dissolution properties was found. Moreover, for the series of FFA-RAMEB the dissolution rate trend was GR > COE > COL >> KN > PM, as shown in Figure 3b. The best dissolution behaviour was obtained with the FFA RAMEB GR (>1600 mg/L after 60 min), probably due to the highest degree of drug amorphization obtained by this technique, so this binary system was selected for tablet formulation.

### 3.4. Preparation and Characterisation of Tablets

The FFA-RAMEB GR product, selected as the binary system that better improved drug solubility, was used to prepare immediate release tablets (IRT), able to give a rapid release and absorption of the drug, for the treatment of acute diseases, and conventional tablets suitable for enteric-coating (CT), more appropriate for the treatment of chronic diseases, that require prolonged drug administration and then the need of minimising the drug irritant effect towards the gastric mucosa. All tablets were prepared by direct compression, which is considered as a more advantageous process than wet granulation in terms of cost effectiveness (lesser processing steps and equipment), higher stability (for moisture- or heat-sensitive drugs), reduced transfer losses and contamination risks, and faster and more reproducible drug dissolution profiles, being this last a particularly beneficial effect for poorly soluble drugs [26,27]. Special excipients, suitable for direct compression, were used. Spray-dried lactose was selected as diluent/filler, while a partially pregelatinised starch was chosen for both disintegrant power and, at the same time, good cohesion and compressibility properties. In the case of tablets intended for enteric coating, PVP K90 was added as a dry binder, to increase the tablets hardness and make them more resistant and less friable.

In order to evaluate the role of the RAMEB presence and of the preparation method used for obtaining the drug-CD binary system, IRT and CT tablets containing the FFA-RAMEB system as GR product were then prepared and compared with analogous tablets containing FFA as such or as simple PM with RAMEB. The obtained tablets were characterised for drug content, mean weight, mean diameter, thickness, hardness, disaggregation time and dissolution properties. The results of these studies are summarised in Table 3 where % dissolved (PD) and dissolution efficiency (DE) are reported at 10, 30 or 60 min. 

As for the technological properties, all manufactured tablets showed good quality attributes, particularly in terms of weight uniformity (RSD < 1%) and drug content uniformity (RSD < 1.3%) [27]. In all cases, adequate disintegration times were observed, which in the case of IRTs were always ≤3 min. The expected higher hardness of CTs was considered suitable to be subjected to the coating process. As for the dissolution properties, as expected, CT presented a lower drug dissolution rate compared to the corresponding IRTs, due to the presence in their formulation of PVPK90, which acting as a binder, increased the tablet’s hardness and, consequently, the disaggregation time. This binder also increases the disaggregation time, due to the difficulty of the solvent to penetrate within the tablet matrix. In both series of tablets, the favourable effect due to the RAMEB presence was evidenced, but, interestingly, those containing the FFA-RAMEB GR system showed dissolution properties, clearly superior to those of the corresponding tablets containing the simple FFA-RAMEB PM, thus confirming the results of dissolution studies on binary systems, and pointing out the important role of the preparation technique of drug-CD solid systems in determining their performance. IRT tablets containing the binary GR systems were the only ones that met the dissolution criterion for immediate release dosage forms, exceeding 90% drug dissolved after 30 min [28].

Previous studies performed on pure drug samples provided from different suppliers (SIMS and TCI) showed that contact angle values of FFA TCI compacts were significantly lower than those of FFA SIMS compacts, indicative of a better wettability of their surface, reflected in a significantly higher intrinsic dissolution rate [13]. Since such a different dissolution behaviour of FFA samples after compaction could be a possible cause of the appearance of bio-inequivalence issues among pharmaceutically equivalent tablets, it was considered worthy of interest to investigate if the differences observed in such previous studies are still present in the new developed tablets. With this aim, CTs were prepared also using FFA from SIMS, and their dissolution behaviour was compared to the corresponding tablets prepared using FFA from TCI.

**Figure 4:**
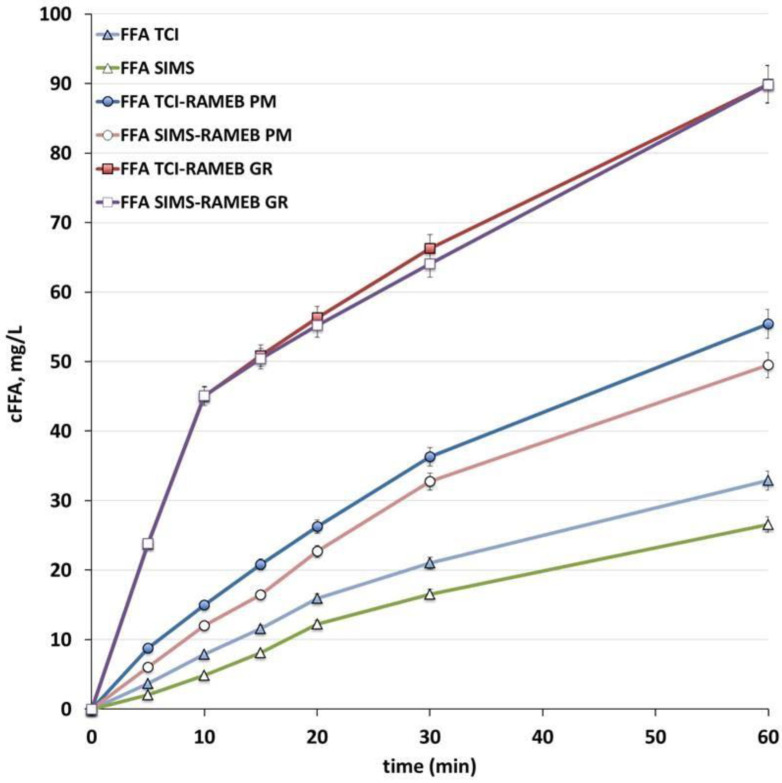
Dissolution profiles of FFA from conventional tablets for enteric coating (CT) containing the drug, provided by TCI or SIMS, as such or as physical mixture (PM) or co-ground product (GR) with RAMEB.

As shown in Figure 4, CTs containing FFA SIMS and FFA SIMS-RAMEB PM exhibited a lower drug dissolution rate compared to the corresponding ones prepared starting from FFA TCI. These results seemed to confirm the worsening of FFA SIMS dissolution properties as a consequence of its compaction [13]. This effect was observed also when it was used as PM with the amorphous RAMEB, where the drug maintained (almost unchanged) its crystalline character, as shown by DSC and XRPD analysis. The different dissolution profiles shown by FFA SIMS and FFA TCI crystalline powders after compaction was attributed to their different behaviours during the compression process, probably related to the different shape and surface properties or their particles [13], thus giving rise to different wettability and density of their compacts. On the contrary, interestingly, these differences disappeared in the case of amorphous FFA powders, being the drug dissolution profiles from tablets containing FFA-RAMEB GR prepared with both FFA batches completely superimposable. These results seemed to suggest that the complete drug amorphization, obtained by co-grinding with RAMEB, not only gave rise to the highest increase in FFA dissolution rate, but also allowed to eliminate any difference in dissolution behaviour between FFA powders coming from different suppliers.

In order to obtain more insight about these results, the role of the tablet surface wettability in the differences observed between tablets obtained with FFA SIMS and the corresponding ones obtained with FFA TCI was then investigated.

**Figure 5:**
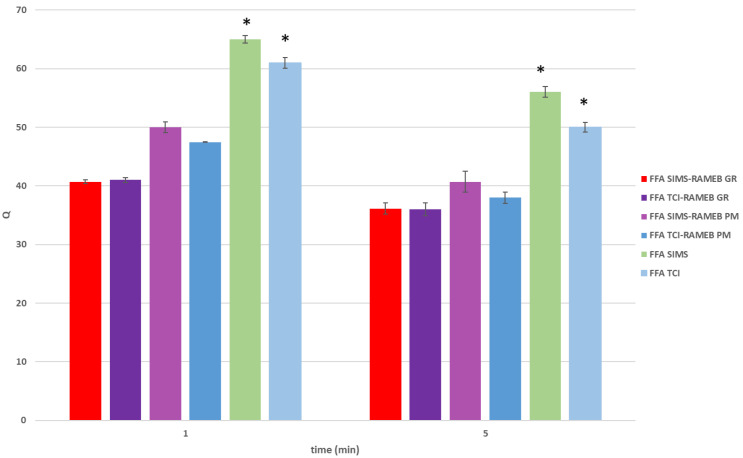
Contact angles (Q) between a water droplet and the surface of the different conventional tablets for enteric coating (CT) containing the drug, provided by TCI or SIMS, as such or as physical mixture (PM) or co-ground product (GR) with RAMEB, at 1 and 5 min after the droplet deposition (mean of 3 determinations). * *p* < 0.05.

The contact angles between a water droplet and CTs containing FFA from SIMS or TCI, alone or as PM or GR with RAMEB were measured and the results are shown in Figure 5.

The presence of RAMEB enhanced the wettability of the tablets in any case due to the high hydrophilicity of this excipient. CTs containing FFA TCI (alone or as PM with RAMEB) showed a wettability significantly better (*p* < 0.05) than those containing FFA SIMS, thus explaining the greater dissolution rate of FFA TCI than FFA SIMS when formulated in tablets. This phenomenon could be attributable to the different surface characteristics of their crystalline powders that led to a higher compactability of FFA SIMS with respect to FFA TCI, as observed in previous study [13]. The highest wettability was observed for tablets obtained with the amorphous GR with RAMEB, independently from the FFA supplier, probably due to the more intimate interactions between the components, and the obtained drug complexation/amorphization.

True density analyses were also performed to further characterise the two series of CT tablets. As can be seen in Table 4, CTs containing FFA TCI crystalline powder, both alone or as PM with RAMEB, showed lower true density values than the corresponding CTs with FFA SIMS, reflecting some higher degree of porosity of their structure. On the contrary, the higher density of tablets containing crystalline FFA-SIMS suggested the greater ability of the particles of this powder, probably related to their rough surface [13], to closely bind each other during the compression process, giving rise to more compact, less porous structures. Interestingly, no differences were instead observed between true density values of CT tablets containing the drug as co-ground product with RAMEB, indicating that complexation/amorphization of FFA obtained in such binary system allowed to eliminate any difference in the compaction behaviour observed in the two original crystalline powder samples provided by the two suppliers. This finding seemed to further corroborate the hypothesis that the above observed differences in the dissolution performance of CT containing FFA as crystalline powder were actually due to differences in their compaction behaviour, resulting in tablets with different surface wettability and different true density.

## 4. Conclusions

This work aimed to develop new tablet formulations with improved dissolution properties of FFA that would allow to overcome the problems of bio-inequivalence, and variable bioavailability shown by commercial dosage forms, related to its poor solubility and dissolution rate. With this purpose, the complexing and solubilising properties towards FFA of different CDs were evaluated, and a series of FFA-CD solid systems was prepared and characterised. Co-grinding with RAMEB was the only technique that allowed the complete drug amorphization, together with the highest improvement in drug dissolution rate, and was then selected for tablets formulation. The FFA-RAMEB co-ground product was formulated as immediate-release tablets (IRTs) or conventional tablets suitable for enteric coating (CTs). A comparison with the corresponding tablets containing the drug as such or as simple physical mixture (PM) with RAMEB evidenced a strong improvement of drug dissolution properties, with an increase, respectively, of 4.0 and 1.6 times (for IRT) or 5.6 and 3.0 times (for CTs) of the percent dissolved at 10 min.

Moreover, in the attempt to gain insight about the unexpected difference in intrinsic dissolution rate constant presented by FFA samples from different suppliers [13], CTs were prepared using FFA from both SIMS and TCI. Even if the two series of tablets showed the same dissolution rate trend (FFA < FFA-RAMEB PM < FFA-RAMEB co-ground), those containing FFA SIMS crystalline powder (both as plain drug or as PM with RAMEB) showed a lower dissolution rate than the corresponding ones containing FFA TCI, confirming the previously observed dissolution differences between the two FFA batches. Wettability and true density studies suggested that such differences were related to a different behaviour of crystalline FFA SIMS or TCI during the compression process, giving rise to compacts with different porosity and surface properties. Interestingly, superimposable dissolution profiles were instead shown by tablets containing the amorphous co-ground product with RAMEB, indicating that FFA complexation/amorphization with such CD allowed not only to remarkably improve its dissolution rate but also to eliminate the differences in dissolution rate among the two drug batches observed in tablets containing the plain drug or its simple PM with RAMEB.

The use of the amorphous co-ground product of FFA with RAMEB enabled to obtain tablets with good and well reproducible dissolution profiles, not affected by the different crystalline properties of the original FFA powder, thus resulting a useful tool for an effective oral administration of this recently rediscovered drug.

## Figures and Tables

**Table 1:** Stability constants (K1:1) and complexation efficiency (CE) of the complexes of flufenamic acid (FFA) with different CDs.

CD	K_1:1_ (M^−1^)	CE (M^−1^)	FFA Solubility Increase *
αCD	32 ± 0.8	34 ± 0.9	1.62
βCD	760 ± 18	815 ± 20	6.22
γCD	n.c.	n.c.	----
HPβCD	1410 ± 35	1511 ± 37	15.43
RAMEB	1860 ± 45	1999 ± 49	17.64
SBEβCD	1812 ± 43	1946 ± 47	16.29

n.c.: not calculated. * ratio between FFA solubility in the presence of the highest CD concentration used and alone.

**Table 2:** Melting temperature (Tm), enthalpy of fusion (∆H) and % residual crystallinity (% Rc) of FFA in its binary systems obtained by physical mixing (PM), kneading (KN), co-grinding (GR), coevaporation (COE) and co-lyophilisation (COL) with βCD, HPβCD, SBEβCD and RAMEB.

Sample	Tm (°C)	ΔH (J/g)	% Rc
FFA	135.4 ± 0.3	124.6 ± 6.0	100.0 ± 4.8
PM FFA βCD	135.2 ± 0.5	115.2 ± 5.7	92.5 ± 4.6
KN FFA βCD	135.1 ± 0.4	82.2 ± 3.9	65.9 ± 3.2
GR FFA βCD	134.8 ± 0.5	35.3 ± 1.7	24.4 ± 1.2
COE FFA βCD	125.4 ± 0.3	74.6 ± 3.6	59.8 ± 2.9
COL FFA βCD	135.2 ± 0.4	27.8 ± 1.3	20.7 ± 1.0
PM FFA HPβCD	135.0 ± 0.4	78.2 ± 3.8	62.8 ± 3.1
KN FFA HPβCD	135.7 ± 0.6	53.3 ± 2.7	42.7 ± 2.1
GR FFA HPβCD	135.8 ± 0.5	25.8 ± 1.3	14.7 ± 0.7
COE FFA HPβCD	125.6 ± 0.4	38.6 ± 1.9	30.9 ± 1.5
COL FFA HPβCD	135.7 ± 0.4	18.6 ± 0.9	13.7 ± 0.6
PM FFA SBEβCD	135.6 ± 0.6	92.8 ± 4.5	74.5 ± 3.6
KN FFA SBEβCD	135.5 ± 0.4	40.8 ± 2.0	32.8 ± 1.6
GR FFA SBEβCD	135.2 ± 0.3	12.2 ± 0.6	9.8 ± 0.5
COE FFA SBEβCD	126.3 ± 0.7	39.5 ± 2.0	31.6 ± 1.6
COL FFA SBEβCD	135.1 ± 0.5	13.7 ± 0.7	10.4 ± 0.5
PM FFA RAMEB	134.2 ± 0.8	29.3 ± 1.5	23.5 ± 1.2
KN FFA RAMEB	n.d.	n.d.	n.d.
GR FFA RAMEB	n.d.	n.d.	n.d.
COE FFA RAMEB	n.d.	n.d.	n.d.
COL FFA RAMEB	n.d.	n.d.	n.d.

n.d.: not detectable.

**Table 3:** Properties of immediate release tablets (IRT) and conventional tablets for enteric coating (CT).

Batch	Drug Content (%)	Mean Weight (mg)	Thickness (cm)	Hardness (Kg/cm^2^)	Disagg. Time (s)	PD 10	PD 30	DE 10	DE 60
IRT									
FFA	100.2 ± 0.9	200 ± 0.7	0.10 ± 0.01	1.5 ± 0.5	100	15.9 ± 0.6	29.6 ± 1.1	7.9 ± 0.3	26.6 ± 1.0
PM	99.9 ± 0.8	433.8 ± 0.9	0.25 ± 0.02	5.0 ± 0.5	160	39.3 ± 1.5	68.5 ± 2.6	22.9 ± 0.8	58.6 ± 2.2
GR	98.9 ± 1.2	433.2 ± 0.5	0.25 ± 0.02	4.0 ± 0.3	180	62.9 ± 2.3	92.8 ± 3.5	38.9 ± 1.5	82.2 ± 3.1
CT									
FFA	99.8 ± 0.8	250.8 ± 0.9	0.20 ± 0.01	15.0 ± 0.5	300	7.9 ± 0.3	21.0 ± 0.7	3.8 ± 0.1	19.1 ± 0.7
PM	98.7 ± 1.3	487.2 ± 0.5	0.30 ± 0.02	17.0 ± 0.3	420	15.0 ± 0.5	36.3 ± 1.3	8.1 ± 0.3	32.9 ± 1.2
GR	99.2 ± 0.9	488 ± 0.4	0.30 ± 0.02	16.5 ± 0.5	450	45.0 ± 1.7	66.3 ± 2.5	23.2 ± 0.8	61.6 ± 2.3

**Table 4:** True density values of the different batches of conventional tablets for enteric coating (CT).

CT	True Density (g/cm^3^)	Coefficient of Variation CV (%)
FFA SIMS	1.426	0.178
FFA TCI	1.379	0.046
FFA SIMS-RAMEB PM	1.431	0.050
FFA TCI-RAMEB PM	1.360	0.040
FFA SIMS-RAMEB GR	1.438	0.029
FFA TCI-RAMEB GR	1.439	0.039

## Data Availability

The shared data on True density performed by Anton Paar are in accordance with the consent on the use of confidential data.

## Supplementary Materials

The following supporting information can be downloaded at: https://www.mdpi.com/article/10.3390/pharmaceutics14020284/s1, Figure S1: DSC thermograms of PM and GR.
