# Supplementary material for: Role of Cyclodextrins and Drug Solid State Properties on Flufenamic Acid Dissolution Performance from Tablets
Source: Pharmaceutics. 2022 Jan 26;14(2):284. doi: 10.3390/pharmaceutics14020284 (PMC8880332; doi:10.3390/pharmaceutics14020284)
Supplement: Supplementary file 1 [file pharmaceutics-14-00284-s001.zip › pharmaceutics-1553182-supplementary.pdf]

pharmaceutics

MDPI

Supplementary Materials: Role of Cyclodextrins and Drug Solid State Properties on Flufenamic Acid Dissolution Performance from Tablets

Francesca Maestrelli, Marzia Cirri, Enrico De Luca, Diletta Biagi and Paola Mura

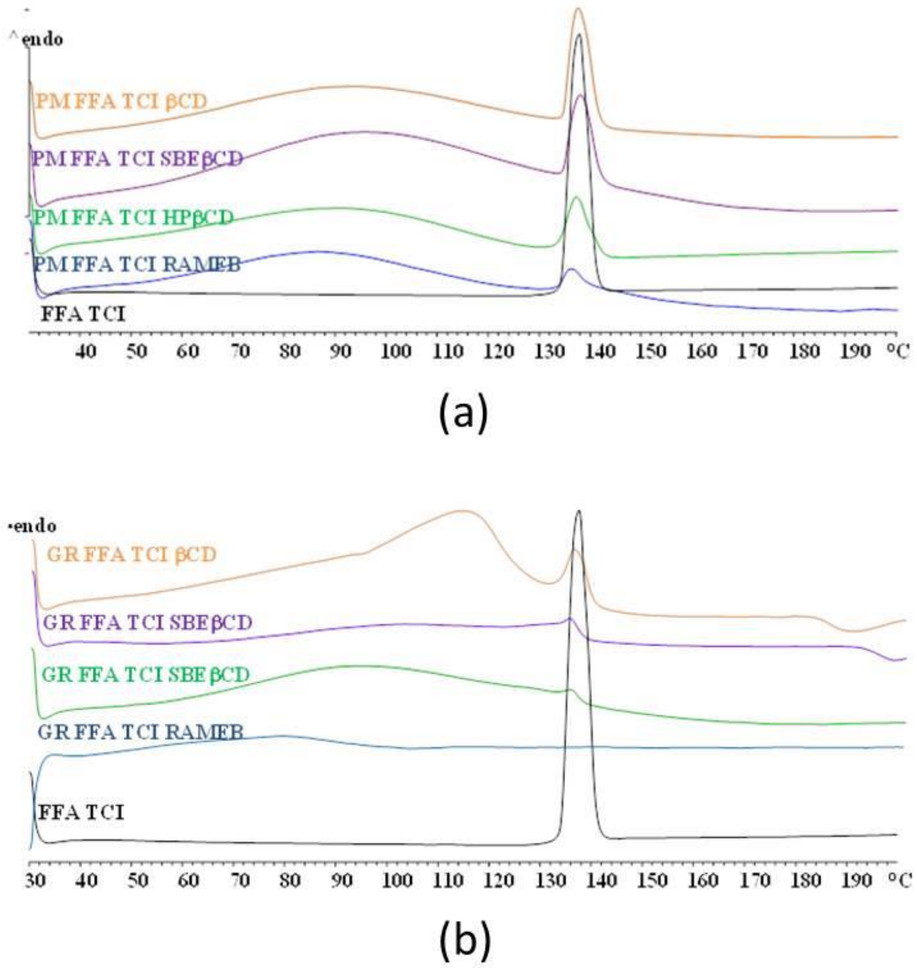

Figure S1. DSC thermograms of PM and GR.
